# Antipsychotic agents deteriorate brain and retinal function in schizophrenia patients with combined auditory and visual hallucinations: A pilot study and secondary follow‐up study

**DOI:** 10.1002/brb3.1611

**Published:** 2020-04-14

**Authors:** Chuanjun Zhuo, Bo Xiao, Ce Chen, Deguo Jiang, Gongying Li, Xiaoyan Ma, Ranli Li, Lina Wang, Yong Xu, Chunhua Zhou, Xiaodong Lin

**Affiliations:** ^1^ Department of Psychiatry Pattern Recognition Laboratory of Schizophrenia School of Mental Health Jining Medical University Jining China; ^2^ Department of Genetics Laboratory of Schizophrenia School of Mental Health Jining Medical University Jining China; ^3^ Department of Psychiatry Wenzhou Seventh People's Hospital Wenzhou China; ^4^ Department of Psychiatric‐Neuroimaging‐Genetics and Co‐morbidity Laboratory (PNGC_Lab) Tianjin Anding Hospital Tianjin Mental Health Center Tianjin Medical University Mental Health Teaching Hospital Tianjin Medical University Tianjin China; ^5^ Department of Psychiatry First Hospital/First Clinical Medical College of Shanxi Medical University Taiyuan China; ^6^ MDT Center for Cognitive Impairment and Sleep Disorders First Hospital of Shanxi Medical University Taiyuan China; ^7^ Department of OCT Tianjin Eye Hospital Tianjin China; ^8^ Department of Pharmacology The First Hospital of Hebei Medical University Shijiazhuang China

**Keywords:** auditory hallucination, gray matter volume, retinal thickness, schizophrenia, vision hallucination

## Abstract

**Introduction:**

Schizophrenia patients often experience auditory hallucinations (AHs) and visual hallucinations (VHs). However, the degree and type of brain and retinal alterations associated with combined AHs and VHs in schizophrenia patients remain unknown. There is an urgent need for a study that investigates the trajectory of brain and retinal alterations in patients with first‐episode untreated schizophrenia accompanied by combined AHs and VHs (FUSCHAV).

**Methods:**

FUSCHAV patients (*n* = 120), divided into four groups according to AH and VH symptom severity (severe AHs combined with severe VHs [FUSCHSASV, 20 patients]; middle‐to‐moderate AHs combined with severe VHs [FUSCHMASV, 23 patients]; severe AHs combined with middle‐to‐moderate VHs [FUSCHSAMV, 28 patients]; and middle‐to‐moderate AHs combined with middle‐to‐moderate VHs [FUSCHMAMV, 26 patients]), were compared to healthy controls (*n* = 30). Gray matter volume (GMV) was adopted for brain structural alteration assessment. Total retinal thickness was adopted as a measure of retinal thickness impairment.

**Results:**

In the pilot study, the rate of GMV reduction showed an inverted U‐shaped pattern across the different FUSCHAV patient groups according to AH and VH severity. The degree of retinal impairment remained stable across the groups. More notably, in the secondary follow‐up study, we observed that, after 6 months of treatment with antipsychotic agents, all the GMV reduction‐related differences across the different patient groups disappeared, and both GMV and retinal thickness demonstrated a tendency to deteriorate.

**Conclusions:**

These findings indicate the need for heightened alertness on brain and retinal impairments in patients with FUSCHAV. Further deteriorations induced by antipsychotic agent treatment should be monitored in clinical practice.

## Graphical abstract

(1) GMV reduction and gFCD increases in the frontal–parietal lobule of FUSCHAV patients demonstrate an inverted U‐shape pattern according to differences in AH and VH severity, indicating functional compensation correlated with volume reduction. (2) There appears to be reciprocal deterioration in auditory and visual disturbances among FUSCHAV patients as observed through both brain and retinal abberations. (3) Although brain alterations in FUSCHAV patients differ with degree of AH and VH severity, retinal impairment remains constant among FUSCHAV groups.

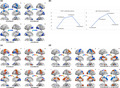

## 1 INTRODUCTION

Auditory perceptual disturbances in schizophrenia patients usually present as auditory hallucinations (AHs) (Pinheiro, Farinha‐Fernandes, Roberto, & Kotz, 2019), and the prevalence of AHs is greater than 40% in such settings (Upthegrove et al., 2016; Zhuo et al., 2019). Auditory hallucinations can be observed at any stage of schizophrenia (Zhuo et al., 2019). In the past 20 years, a large number of studies have investigated AHs and put forth several related hypotheses focusing on the pathological mechanism of AHs (Baumeister, Sedgwick, Howes, & Peters, 2017; Blom, 2015; Hugdahl & Sommer, 2018; Northoff, 2014). Those studies provided many important findings and pivotal clues for the investigation of the brain‐related mechanisms behind AHs in schizophrenia patients (Huang, Zhuo, Xu, & Lin, 2019; Stephane, 2019; Zmigrod, Garrison, Carr, & Simons, 2016).

Visual disturbances in patients with schizophrenia usually present as visual hallucinations (VHs) or vision distortions (Green, Hellemann, Horan, Lee, & Wynn, 2012). The prevalence of VHs in schizophrenia patients is 25%–30% (Waters et al., 2014). Many previous studies have reported that VHs can be observed in any stage of schizophrenia (including the prodromal stage, acute stage, and chronic stage). In addition, VHs have also been observed in children, adolescents, and young adults at a high risk for schizophrenia (Garcia‐Portilla et al., 2019; Grano et al., 2015; Guidotti & Grayson, 2011; Hebert et al., 2010; Mittal, Gupta, Keane, & Silverstein, 2015). Some studies that adopted neuroimaging and neuro‐electrophysiology techniques reported that VHs are usually accompanied by brain cortex‐related functional and structural alterations (Henke, Robinson, Drysdale, & Loxley, 2014; Pajani, Kok, Kouider, & Lange, 2015; Stephan‐Otto et al., 2017). Most of those studies reported that functional disturbances in the visual cortex and parietal cortex, and structural alterations in the brain gray matter volume (GMV) were characteristic of VHs (De Haan, Nys, Zandvoort, & Ramsey, 2007; Garcia‐Portilla et al., 2019; Grano et al., 2015; Guidotti & Grayson, 2011; Hebert et al., 2010; Henke et al., 2014; Mittal et al., 2015; Oertel et al., 2007; Pajani et al., 2015; Stephan‐Otto et al., 2017). Several previous studies have reported that VHs are also accompanied by visual perception organization‐related impairments, especially retinal thickness impairments (Lee et al., 2014; Schönfeldt‐Lecuona et al., 2019). In recent years, an increasing number of studies have been using optical coherence tomography (OCT) for the investigation of the retinal thickness‐related alterations in schizophrenia patients (Calderone et al., 2013; Lencer, Nagel, Sprenger, Heide, & Binkofski, 2005; Nagel et al., 2007; Onitsuka et al., 2007; Silverstein et al., 2009, 2010); however, few studies have focused on the retinal thickness‐related impairments in schizophrenia patients with VHs. There is an urgent need for studies to be conducted to bridge this gap and explore the mechanisms of VHs in schizophrenia patients.

AHs and VHs can co‐occur in patients with schizophrenia (Andreasen & Flaum, 1991; van Os & Kapur, 2009), and such a co‐occurrence is usually associated with an increased difficulty in treatment administration and prognostic deterioration (Elkis & Buckley, 2016; Potkin et al., 2020). However, to the best of our knowledge, few studies have reported on the brain‐related features of schizophrenia patients with VH and AH co‐occurrence. A previous study demonstrated that the brain functional connectivity‐related disturbances in the mesolimbic pathway, especially in the nucleus accumbens, were characteristic of AH and VH co‐occurrence in schizophrenia patients (Rolland et al., 2015). The findings of that study provide important clues for the re‐consideration of the brain‐related features in such settings. Auditory hallucinations are usually accompanied by functional disturbances in the components of the speech network or salience network (Jardri, Pouchet, Pins, & Thomas, 2011). Visual hallucinations are accompanied by functional disturbances mainly in the visual cortex (Behrendt, 2010) and hippocampus (Amad et al., 2014). Schizophrenia patients with the co‐occurrence of VHs and AHs demonstrate functional connectivity‐related disturbances in the mesolimbic pathway (Rolland et al., 2015). These findings indicate that, in schizophrenia patients, AH and VH co‐occurrence may be accompanied by more complex in‐brain functional and structural alterations. However, although the aforementioned studies provide information on the brain‐related pathological features of schizophrenia patients with AH and VH co‐occurrence, several questions remain unanswered. For example, (a) Are the different categories of AH and VH co‐occurrence severity in schizophrenia patients associated with different brain‐related structural and functional alterations? (b) Do the retinal thickness‐related impairments differ across the different VH and AH combination categories in such patients? (c) Will the VHs and AHs deteriorate reciprocally? and (d) Will the antipsychotic agents used for treatment influence the brain and retinal alterations in such settings?

Based on the findings of previous studies (Amad et al., 2014; Rolland et al., 2015; Silverstein, 2016), especially those obtained by Silverstein (Silverstein, 2016), we conducted a study to investigate the pathological features associated with first‐episode unmedicated schizophrenia with combined auditory and visual hallucinations (FUSCHAV). Unlike prior studies that categorized patients by their experience of either AHs or VHs, in the current study, we investigated all the brain and retinal aberrations presenting in those with a combination of AHs and VHs. We hypothesized that: (a) FUSCHAV would be associated with brain structural/functional and retinal alterations, (b) The brain and retinal alteration patterns may differ across different AH‐VH combination severity categories, and (c) AHs and VHs may show reciprocal deterioration in these patients.

## 2 METHODS

Patients were recruited for participation, between January 2018 and July 2019, from five institutes (Tianjin Mental Heal Center, Tianjin Anding Hospital, the School of Mental Health at Jining Medical University, Wenzhou Seventh People's Hospital, Tianjin Kangtai Hospital, and The First Hospital of Shanxi Medical University). The ethics committee of all five institutes approved the study methods. All magnetic resonance imaging (MRI) and OCT studies were conducted by one MRI scanner type (Discovery MR750, General Electric) and OCT system type (OCT 4000 system, Zeiss, premierop.com/zeiss‐cirrus‐hd‐oct‐4000‐used), respectively. The MRI and OCT parameters in four institutes (Tianjin Anding Hospital and Tianjin Kangtai Hospital used one MRI and OCT system) were unified by a professional MRI technician and ophthalmologist to ensure the highest consistency.

The inclusion criteria for FUSCHAV were as follows: (a) complete fulfillment of Diagnostic and Statistical Manual of Mental Disorders, fourth edition (DSM‐IV) criteria for schizophrenia, (b) first schizophrenia diagnosis at a mental health hospital, (c) absence of antipsychotic therapy use for at least 3 weeks prior to the study, (d) simultaneous experience of AHs and VHs, (e) age 18–30 years, (f) absence of substance abuse, (g) absence of other systemic disease, chronic disease, or head trauma, (h) absence of other diseases that may cause AHs and VHs, and (i) absence of other diseases that can cause retinal disease. Healthy controls were recruited from among hospital staff and adult medical students. The healthy controls did not exhibit psychiatric disorders or have first‐degree relatives with psychotic disorders, as assessed by two professional psychiatrists using the Structured Clinical Interview for DSM‐IV‐Non‐patient edition. The exclusion criteria for the patients and healthy controls were as follows: (a) presence of moderate‐to‐severe physical disease (e.g., respiratory, cardiovascular, endocrine, neurological, liver, or kidney disease); (b) current reception of electroconvulsive therapy; (c) history of a loss of consciousness for more than 5 min from any cause; (d) left‐handedness, as determined by the Annett Hand Preference Questionnaire (Dragovic & Hammond, 2007); (e) ophthalmic disease; (f) high myopia; (g) any MRI contraindication, including claustrophobia; and (h) intelligence quotient <80. The healthy controls did not have a positive family history, as assessed by the Family Interview for Genetic Studies (1992).

### 2.1 MRI data acquisition

The 3.0‐Tesla MR system (Discovery MR750, General Electric) was used for the collection of MRI data. Functional MRI (fMRI) was performed using a GE Healthcare Discovery MR750 3 T MRI system (General Electric) with an eight‐channel phased‐array head coil. Participants lay in a supine position and were asked to restrict their thoughts and head movements during imaging. The imaging parameters were as follows: 2,000 ms repetition time (TR), 45 ms echo time (TE), 32 slices, 4 mm slice thickness, 0.5 mm gap, field of view, 64 × 64 acquisition matrix, and 90° flip angle. SENSitivity Encoding (SENSE) with a SENSE factor of 2 and parallel imaging were used for all scans. Images were obtained with a high‐resolution, three‐dimensional turbo‐fast echo T1‐weighted sequence with the following parameters: 8.2/3.2‐ms TR/TE, 188 slices, 1 mm thickness, no gap, 256 × 256 FOV, 256 × 256 acquisition matrix, and 12° FA.

### 2.2 OCT data acquisition

OCT data were acquired with the OCT 4000 system (Zeiss [premierop.com/zeiss‐cirrus‐hd‐oct‐4000‐used]) that collects retinal scans of both eyes. The hand‐held probe was mounted, and participants were positioned on a chin‐head rest and asked to focus on a fixed target. A 5‐s volumetric 10 mm × 5 mm scan of the foveal center, marked by outer segment layer thickening, was captured. Each scan comprised 500 A‐scan/B‐scans, 50 B‐scans, and five frames/B‐scan, with acceptable scans containing ≥ five consecutive B‐scan frames of the foveal center with no movement artefacts.

### 2.3 Psychotic, auditory, and visual hallucination symptom severity assessment

The total severity of schizophrenia was assessed by the Positive and Negative Symptoms Scale (PANSS) (Fleischhacker et al., 2019). The Scale for the Assessment of Positive Symptoms (SAPS) (Kumari et al., 2017) was used to measure the severity of AHs and VHs. Cognitive ability was assessed by the MATRICS Consensus Cognitive Battery (MCCB) (Lees et al., 2015). The Global Assessment of Functioning (GAF) was used to assess global function (Gspandl, Peirson, Nahhas, Skale, & Lehrer, 2018).

Patients were categorized into four groups according to AH and VH severity, as measured by the SAPS items of VH and AH. Group 1 presented with severe AHs and VHs (FUSCHSASV, *n* = 20), Group 2 with middle‐to‐moderate AHs combined with severe VHs (FUSCHMASV, *n* = 23), Group 3 with severe AHs combined with middle‐to‐moderate VHs (FUSCHSAMV, *n* = 28), and Group 4 with middle‐to‐moderate AHs combined with middle‐to‐moderate VHs (FUSCHMAMV, *n* = 26).

### 2.4 Structural MRI data processing

The analysis of brain volume differences was performed by voxel‐based morphometry, using SPM8 (Statistical Parametric Mapping; v5; Institute of Neurology, London, UK). Bias correction, spatial normalization, segmentation into gray and white matter, imaging of cerebrospinal fluid, and intensity modulation were performed on 3D‐FSPGR images in native space using SPM8. The Diffeomorphic Anatomical Registration Through Exponential Lie Algebra toolbox, as proposed by Ashburner, was used following a high‐dimensional normalization protocol. Intensity modulation was performed by multiplying the voxel values of the segmented images by the measure of warped and unwarped structures derived from the nonlinear step of the spatial normalization. During this step, relative regional gray matter density was converted into absolute gray matter density and expressed as the amount of gray matter per unit volume of brain tissue before spatial normalization. The resulting modulated gray and white matter images were smoothed with a 6‐mm Gaussian kernel. Multiple pattern recognition analysis was used to regress out the covariates' influence on gray matter. The significance threshold was set at an FWE‐corrected *p* value <.05. Covariates included age, sex, education level, symptoms severity, GAF score, and MCCB score.

### 2.5 fMRI data preprocessing

Resting‐state fMRI scans were processed using Statistical Parametric Mapping 8 (SPM8; http://www.fil.ion.ucl.ac.uk/spm). The first 10 scan volumes were discarded to allow stabilization of the scanner and to allow patients to get acclimatized to the testing situation. The remaining volumes were corrected for slice timing and motion artifacts. Allowable motion thresholds (translational and rotational motion <2 mm and 2°, respectively) were checked for all fMRI data. Six of the motion parameters and the average blood oxygen level‐dependent signals of the ventricles and white matter were removed. Data with specific‐volume framewise displacement values >0.5 were excluded from the analysis. Bandpass frequencies ranging from 0.01 to 0.08 Hz were used for data filtration. Individual structural images were co‐registered to the mean functional image, and the transformed structural images were co‐registered to the Montreal Neurological Institute (MNI) space using linear registration. The motion‐corrected functional volumes were spatially normalized to the MNI using parameters estimated during linear co‐registration. Finally, the functional images were re‐sampled into 3‐mm cubic voxels for further analysis.

### 2.6 Global Functional Connectivity Density (gFCD) calculation

The gFCD was calculated for each voxel using a customized Linux script (Zhuo et al., 2014). Pearson's linear correlation was used to explore the functional connectivity between voxels, with a correlation coefficient threshold of *r* > .6. Only voxels within the cerebral gray matter mask were used in the calculation of gFCD, and the gFCD for any given voxel (*x*0) was calculated as the total number of functional connections [*k*(*x*0)] between *x*0 and all other voxels using a growth algorithm. This procedure was repeated for all voxels. To increase the normality of the distribution, each gFCD value was divided by the mean value of all the included voxels. A 6 × 6 × 6 mm^3^ Gaussian kernel was used to spatially smooth the gFCD maps to minimize the impact of anatomical differences between the participants (Zhuo et al., 2014).

### 2.7 OCT data analysis

A blind process of manual segmentation of individual retinal layers was performed by allocating random numbers to B‐scan images prior to analysis. An ImageJ macro (http://imagej.nih.gov/ij/) (Jerotic et al., 2019) was used for segmentation. Average individual and combined layer thickness measurements were extracted from three macular regions relative to the foveal center (0 µm): (a) foveal region = −750–750 µm, (b) nasal parafoveal region = −1,500 to −750 µm, and (c) temporal parafoveal region = 750–1,500 µm.

### 2.8 Statistical analyses

One‐way analyses of variance were used to analyze the participants' demographic and clinical characteristics (McHugh, 2011). The Mann–Whitney *U* test was used to compare the GMV and gFCD values between the groups (Matsouaka & Betensky, 2015). Sex‐related differences in terms of the GMV and gFCD corrected were explored using a chi‐square test between the two groups (Thomas, 1990). A *p*‐value <.05 was considered significant.

## 3 RESULTS

### 3.1 Demographic and clinical characteristics of the participants

MRI data from five patients and OCT data from two patients were excluded from the analysis due to poor acquisition quality. Of the remaining 113 patients, 109 demonstrated brain gray matter reductions, and 97 showed brain and retinal co‐structural impairment. Data from these 97 patients were used for the analysis. Patients were divided into four groups according to their SAPS‐AH and SAPS‐VH scores. Score greater than 4 denoted greater severity. Patients with scores between 2 and 3 were defined as having middle‐to‐moderately severe AHs and VHs. According to the above‐mentioned classification rule, we established four groups ([FUSCHSASV, 20 patients]; [FUSCHMASV, 23 patients]; [FUSCHSAMV, 28 patients]; and [FUSCHMAMV, 26 patients]). While the patient groups significantly differed from the healthy controls in terms of the MCCB and GAF scores, there were also significant differences in these measures across the four patient groups (Table 1).

**TABLE 1:** Participants' demographic and clinical characteristics

Variable	HCs *n* = 30	FUSCH SASV *n* = 20	FUSCH MASV *n* = 23	FUSCH SAMV *n* = 28	FUSCH MAMV *n* = 26	*F*	*p*
Age, years Mean (*SD*)	25.4 (0.5)	22.0 (4.2)	26.4 (3.0)	25.2 (1.2)	27.9 (3.9)	63.40	<.001
Sex (female/male)	15/15	9/11	11/12	14/14	11/15	24.23	<.001
Education level, years Mean (*SD*)	16.1 (2.5)	14.5 (2.0)	15.0 (3.5)	14.5 (2.5)	15.5 (1.5)	23.12	<.001
Duration of illness Months, Mean (*SD*)	N/A	2.4 (1.8)	3.2 (2.0)	4.3 (1.5)	6.2 (2.7)	47.21	<.001
PANSS score Mean (*SD*)	N/A	78.9 (1.5)	80.1 (6.8)	79.5 (5.9)	78.6 (9.9)	127.73	<.001
SAPS‐AHs	N/A	4.6 (0.2)	2.2 (0.4)	4.5 (0.1)	1.9 (0.4)	27.73	<.001
SAPS‐VHs	N/A	4.2 (0.3)	3.7 (0.2)	2.0 (0.5)	2.1 (0.3)	21.56	<.001
GAF score Mean (*SD*)	100.0 (0.0)	78.0 (13.5)	72.0 (10.5)	76.5 (6.7)	80.2 (9.9)	45.13	<.001
MCCB score Mean (*SD*)		186.5 (15.2)	211.3 (25.7)	237.1 (29.0)	242.8 (25.8)		
Speed processing	48.0 (4.5)	30.1 (8.5)	36.5 (7.0)	38.5 (8.0)	40.3 (8.0)	99.66	<.001
Attention	47.5 (11.5)	20.3 (4.3)	22.0 (2.7)	23.8 (6.5)	34.0 (2.0)	100.10	<.001
Working memory	50.0 (11.2)	21.2 (4.6)	24.0 (5.2)	34.0 (4.3)	30.2 (8.2)	98.96	<.001
Verbal learning	49.50 (5.0)	30.4 (5.5)	33.2 (9.4)	37.4 (2.5)	32.2 (8.4)	77.52	<.001
Visual learning	45.0 (9.0)	24.0 (3.3)	29.2 (4.8)	30.0 (4.1)	32.2 (2.3)	111.10	<.001
Problem reasoning	45.6 (4.5)	30.0 (7.3)	32.0 (6.5)	35.5 (7.3)	37.0 (10.2)	93.89	<.001
Social cognition	47.5 (1.5)	30.5 (10.0)	34.4 (8.2)	33.4 (8.5)	36.9 (12.0)	112.99	<.001

Abbreviations: AH, auditory hallucination; FUSCHAV, first‐episode untreated schizophrenia with combined AHs and VHs; FUSCHMAMV, middle‐to‐moderate AHs combined with middle‐to‐moderate VHs; FUSCHMASV, middle‐to‐moderate AHs combined with severe VHs; FUSCHSAMV, severe AHs combined with middle‐to‐moderate VHs; FUSCHSASV, severe AHs combined with severe VHs; GAF, Global Assessment of Functioning; HC, healthy control; MCCB, MATRICS Consensus Cognitive Battery; PANSS, Positive and Negative Symptoms Scale; SAPS, Scale for the Assessment of Positive Symptoms; *SD*, standard deviation; VH, visual hallucination.

### 3.2 GMV reduction

**FIGURE 1:**
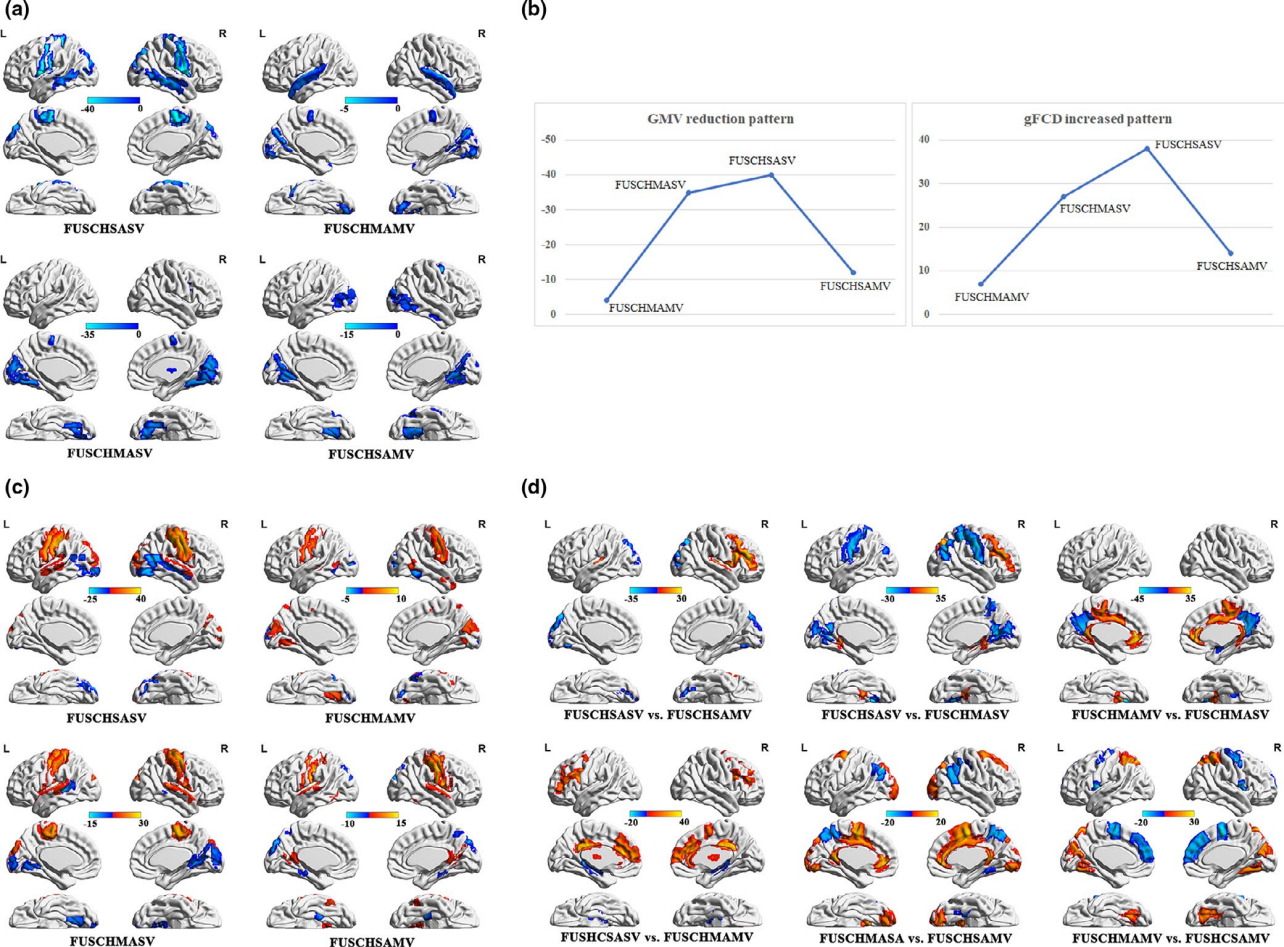
Patients' GMV and gFCD. (a) Location of GMV reduction by patient group. (b) The inverted U‐shape pattern of the GMV and gFCD alterations. *X*‐axis, types of disease combination; *Y*‐axis, mean value. (c) gFCD alterations in FUSCHAV patients. (d) Alterations in the GMV among the patient groups. FUSCHAV, first‐episode untreated schizophrenia with combined AHs and VHs; gFCD, global functional connectivity density; GMV, gray matter volume

Compared to the healthy control group, all four patient groups demonstrated GMV reductions, which were predominantly observed in the temporal, frontal, parietal, and occipital lobes, with the greatest extent of GMV reduction observed in the frontal‐parietal lobe across all four groups (Figure 1a). The four groups, ranked by the extent of GMV reduction according to the peak value of reduction in the frontal‐parietal lobe, were as follows: FUSCHSASV, FUSCHMASV, FUSCHSAMV, and FUSCHMAMV. The extent of GMV reduction demonstrated an inverted U‐shaped pattern (Figure 1b).

### 3.3 gFCD alterations

Compared to the healthy control group, all four patient groups demonstrated gFCD increases. As in the case of GMV alterations, the increase in the gFCD was mainly observed in the temporal, frontal, parietal, and occipital lobes (Figure 1c), with the greatest gFCD increase extent noted in the frontal‐parietal lobe across all four patient groups. The four groups, ranked by the gFCD increase extent in the frontal‐parietal lobe according to peak value (greatest increase to lowest), were as follows: FUSCHSASV, FUSCHMASV, FUSCHSAMV, and FUSCHMAMV. As in the case of the GMV decrease extent, the extent of the gFCD increase demonstrated an inverted U‐shaped pattern (Figure 1b).

### 3.4 GMV and gFCD differences among the patient groups

Alterations in the GMV were compared across the patient groups (Figure 1d). Although the extent of the GMV and gFCD decreases in some brain regions differed between patient groups, and the GMV and gFCD even increased to a greater degree in some patient groups than others, the largest decrease in the scope of GMV and gFCD alterations in comparison with that in the control group remained in the frontal and parietal lobes. This indicates that GMV/gFCD alterations in these regions are uniform pathological features of FUSCHAV.

### 3.5 Retinal alterations

**FIGURE 2:**
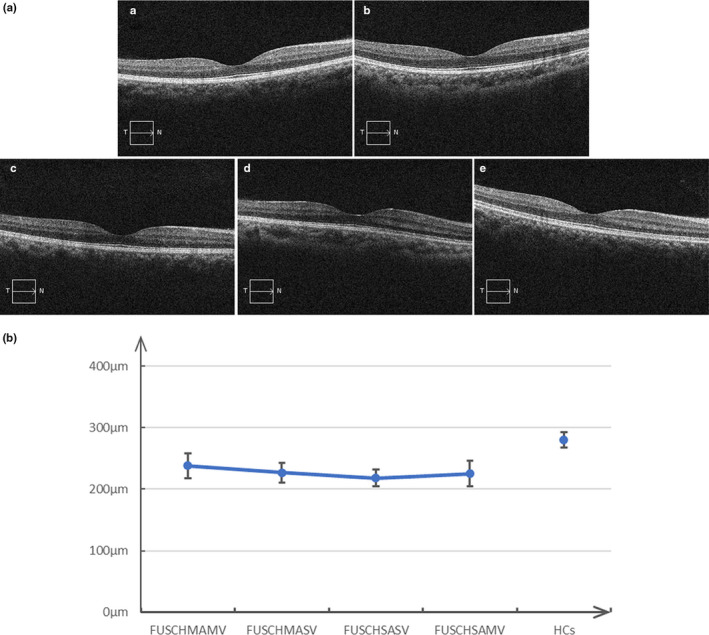
Total retinal thickness and pattern of retinal thinning in patients and healthy controls. (A) The maximum total retinal thickness impairment in the FUSCHSASV (a), FUSCHMASV (b), FUSCHSAMV (c), and FUSCHMAMV (d) groups, and the minimum total retinal thickness of the healthy controls (e). (B) The pattern of retinal thinning in patients and healthy controls. FUSCHAV, first‐episode untreated schizophrenia with combined AHs and VHs; FUSCHMAMV, middle‐to‐moderate AHs combined with middle‐to‐moderate VHs; FUSCHMASV, middle‐to‐moderate AHs combined with severe VHs; FUSCHSAMV, severe AHs combined with middle‐to‐moderate VHs; FUSCHSASV, severe AHs combined with severe VHs

While all four patient groups showed significant retinal thinning compared to the healthy control group, there were no significant differences among the four patient groups in retinal thickness (Figure 2 and Table 2).

**TABLE 2:** Total retinal thickness alterations among different groups on OCT

Variable	Temporal parafoveal region	Foveal region	Nasal parafoveal region
FUSCHSASV	276.0 µm ± 15.0 µm	250.9 µm ± 20.9 µm	310.3 µm ± 10.7 µm
FUSCHMASV	280.3 µm ± 28.3 µm	253.5 µm ± 15.0 µm	312.2 µm ± 14.5 µm
FUSCHSAMV	274.0 µm ± 19.5 µm	257.1 µm ± 16.5 µm	317.2 µm ± 25.4 µm
FUSCHMAMV	275.5 µm ± 10.0 µm	254.5 µm ± 12.0 µm	310.5 µm ± 10.7 µm
Healthy controls (HCs)	315.5 µm ± 15.0 µm	268.3 µm ± 25.0 µm	330.5 µm ± 11.5 µm
Four FUSCHAV groups versus HCs			
*F*	17.56	21.03	30.03
*p*	<.001	<.001	<.001
FUSCHSASV versus FUSCHMASV			
*t* test	0.757	0.720	0.580
*p*	.456	.780	.611
FUSCHSASV versus FUSCHSAMV			
*t* test	0.605	0.742	0.647
*p*	.576	.631	.599
FUSCHSASV versus FUSCHMAMV			
*t* test	0.553	0.653	0.580
*p*	.428	.524	.413
FUSCHMASV versus FUSCHMAMV			
*t* test	0.620	0.753	0.673
*p*	.413	.786	.527
FUSCHMASV versus FUSCHSAMV			
*t* test	0.703	0.603	0.499
*p*	.670	.570	.553
FUSCHSAMV versus FUSCHMAMV			
*t* test	0.996	0.569	0.666
*p*	.878	.578	.591

Abbreviations: FUSCHAV, first‐episode untreated schizophrenia with combined AHs and VHs; FUSCHMAMV, middle‐to‐moderate AHs combined with middle‐to‐moderate VHs; FUSCHMASV, middle‐to‐moderate AHs combined with severe VHs; FUSCHSAMV, severe AHs combined with middle‐to‐moderate VHs; FUSCHSASV, severe AHs combined with severe VHs; HC, healthy control; OCT, optical coherence tomography.

## 4 AIM OF THE SECONDARY FOLLOW‐UP STUDY

In the pilot study, we observed that the GMV and total retinal thickness reduced in cases with FUSCHAV. We also noted a compensatory gFCD increase in these patients. Compared to the healthy controls, we observed serious GMV and retinal thickness reductions in the FUSCHAV patients. Previous studies have reported that antipsychotic agent treatment deteriorates the rate of GMV reduction within the 2–3 years after acceptance. However, few studies have focused on whether retinal thickness deteriorates with antipsychotic agent administration; this question is also the research hot spot proposed by Silverstein (2016). Based on our pilot study and the findings of previous studies, pertaining to the effect of antipsychotic agents on the brain and retina, we attempted to conduct a secondary follow‐up study to clarify the relationship between antipsychotic agent treatment and brain and retinal alterations in the FUSCHAV patients. In the secondary follow‐up study, we hypothesized that (a) brain and retinal alterations will be accompanied by antipsychotic agent use; (b) brain and retinal alterations in the four different FUSCHAV categories may demonstrate different aberrant patterns.

## 5 SECONDARY FOLLOW‐UP STUDY PROCEDURE

In the secondary follow‐up study, the follow‐up time was set at 6 months after the administration of regular antipsychotic agents. The PANSS and SAPS scales were used to assess treatment effect. GMV, gFCD, and total retinal thickness were adopted to assess the effect of antipsychotic agent treatment on the brain and retina in the FUSCHAV patients. The MRI scanner, OCT system, and the parameters and analysis methods employed were the same as those used in the pilot study.

## 6 RESULTS OF THE SECONDARY FOLLOW‐UP STUDY

After the 6‐month treatment period, the PANSS sores reduced by 28.4% in the FUSCHSASV group, 33.7% in the FUSCHSAMV group, 42.9% in the FUSCHMASV group, and 45.0% in the FUSCHMAMV group. The SAPS‐AH scores reduced by 36.9% in the FUSCHSASV group, 40.0% in the FUSCHSAMV group, 45.4% in the FUSCHMASV group, and 52.6% in the FUSCHMAMV group. The SAPS‐VH scores reduced by 13.0% in the FUSCHSASV group, 15.0% in the FUSCHSAMV group, 16.2% in the FUSCHMASV group, and 23.8% in the FUSCHMAMV group. The MCCB total score reduction rate was 15.0% in the FUSCHSASV group, 14.2% in the FUSCHSAMV group, 14.1% in the FUSCHMASV group, and 22.0% in the FUSCHMAMV group. The GAF total score reduction rate was 7.9% in the FUSCHSASV group, 11.5% in the FUSCHSAMV group, 12.8% in the FUSCHMASV group, and 25.7% in the FUSCHMAMV group (Table 3).

**TABLE 3:** Effect of antipsychotic agent treatment

Variable	FUSCH SASV *n* = 20	FUSCH MASV *n* = 23	FUSCH SAMV *n* = 28	FUSCH MAMV *n* = 26	*F*	*p*
Total dosage of chlorpromazine equivalent during the 6‐month treatment period (g)	112.5 (27.9)	104.9 (20.5)	115.2 (30.5)	95.6 (10.0)	58.55	<.001
PANSS score Mean (*SD*)	56.5 (4.2)	45.7 (9.1)	52.7 (8.0)	43.2 (12.1)	55.68	<.001
SAPS‐AHs	2.9 (0.5)	1.2 (0.2)	2.7 (0.4)	0.9 (0.1)	43.28	<.001
SAPS‐VHs	3.6 (0.5)	3.1 (0.5)	1.7 (0.52)	1.6 (0.1)	36.77	<.001
MCCB total score Mean (*SD*)	158.5 (9.2)	181.5 (10.7)	203.4 (18.7)	189.4 (17.5)	37.02	<.001
GAF score Mean (*SD*)	71.8 (19.8)	62.8 (11.0)	67.7 (10.9)	59.6 (9.8)	63.00	<.001

Abbreviations: AH, auditory hallucination; FUSCHAV, first‐episode untreated schizophrenia with combined AHs and VHs; FUSCHMAMV, middle‐to‐moderate AHs combined with middle‐to‐moderate VHs; FUSCHMASV, middle‐to‐moderate AHs combined with severe VHs; FUSCHSAMV, severe AHs combined with middle‐to‐moderate VHs; FUSCHSASV, severe AHs combined with severe VHs; GAF, Global Assessment of Functioning; MCCB, MATRICS Consensus Cognitive Battery; PANSS, Positive and Negative Symptoms Scale; SAPS, Scale for the Assessment of Positive Symptoms; *SD*, standard deviation; VH, visual hallucination.

**FIGURE 3:**
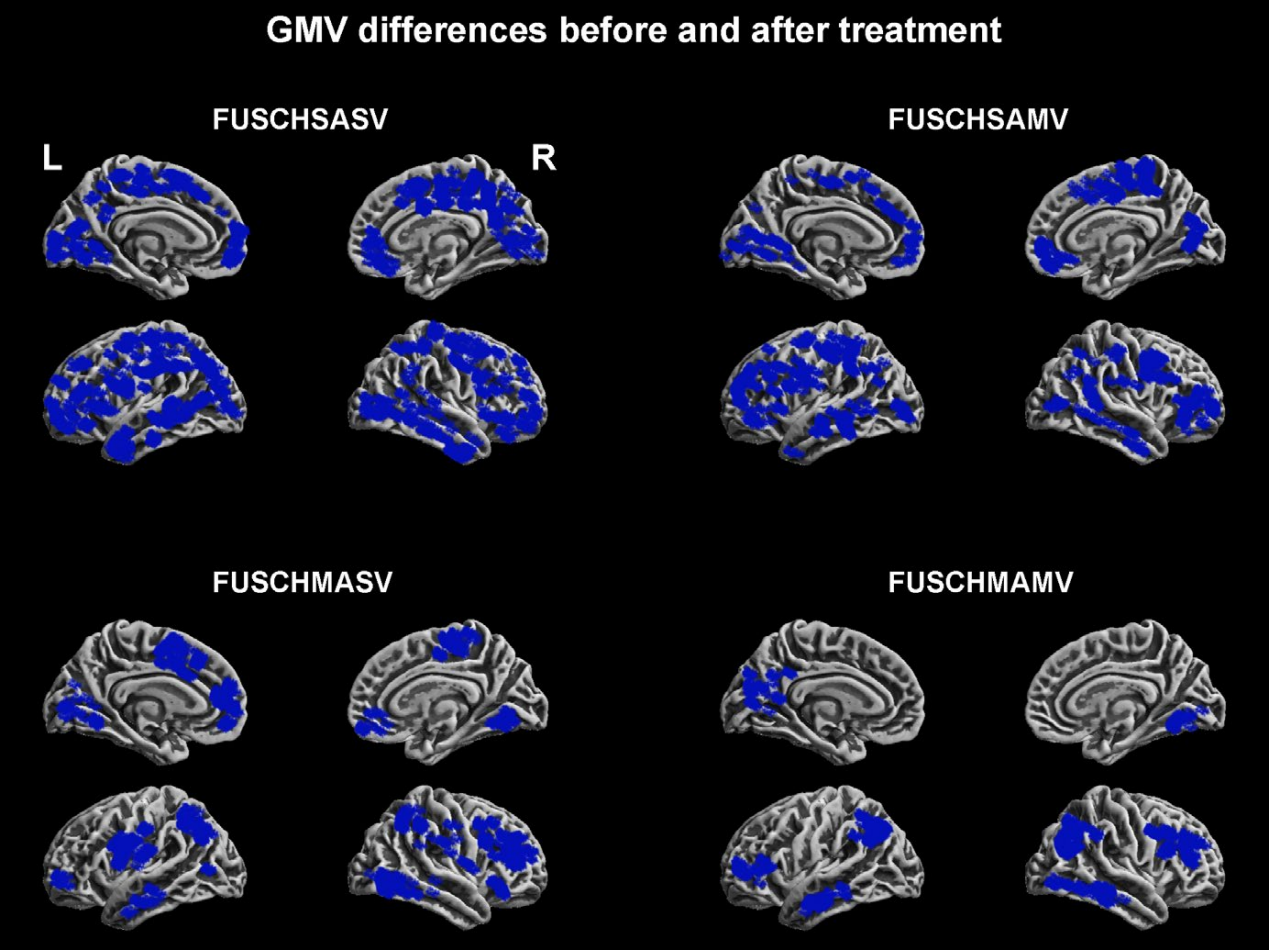
GMV reductions after antipsychotic agent use. GMV, gray matter volume

**FIGURE 4:**
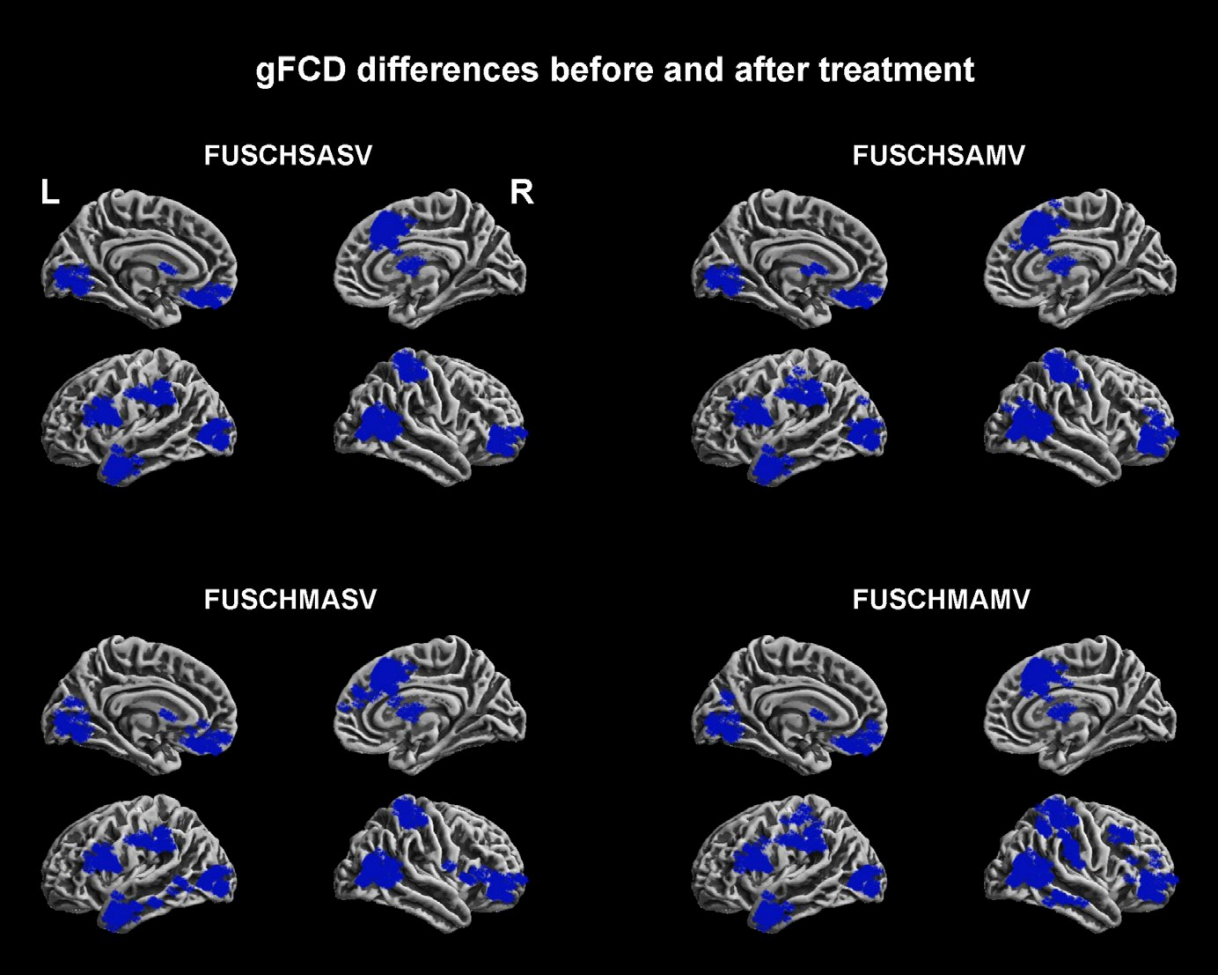
gFCD reductions after antipsychotic agent use. gFCD, global functional connectivity density

In the secondary follow‐up study, we observed that the GMV and gFCD sharply decreased (Figures 3 and 4). The maximum GMV reduction scope was observed in the FUSCHSASV group, followed by the FUSCHSAMV, FUSCHMASV, and FUSCHMAMV groups (Figure 3). The maximum gFCD reduction scope was observed in the FUSCHSAMV group, followed by the FUSCHMASV, FUSCHMAMV, and FUSCHSASV groups. More notably, no significant differences were observed in the degree of GMV reduction between the four groups (a *t* test was used to compare two groups). However, the gFCD did not show significant differences across the groups (a *t* test was used to compare two groups) (Figure 4). The inverted U‐shaped pattern of the GMV and gFCD increase we observed at the baseline disappeared. The maximum GMV reduction rates compared to those at the baseline were 1.37% (self‐comparison) and 1.93 (compared to that in the healthy controls at the baseline); this value was observed in the FUSCHSASV group.

More interestingly, although we observed that the GMV reduction rate was correlated with the total dosage of chlorpromazine equivalent in the FUSCHSASV and FUSCHSAMV groups, the correlation coefficient was very small (*r* = .0023, *p* < .001; *r* = .0017, *p* < .001, respectively). We believe that this correlation was nonsignificant, as, according to statistical theory, *r* < .2 usually denotes that the correlation is delicate (Burger & Missov, 2016; Mbuagbaw et al., 2013).

In this secondary follow‐up study, the total retinal thickness deteriorated after 6 months of treatment with antipsychotic agents. The maximum total thickness decrease was observed in the FUSCHSAMV group, followed by the FUSCHSASV, FUSCHMASV, and FUSCHMAMV groups. The maximum reduction rate was 14.8% (self‐compared, compared to that at the baseline) and 20.0% (compared to that in the healthy controls at the baseline) (Table 4). The region showing the maximum reduction was the nasal parafoveal region, as noted in the FUSCHSAMV group. No significant correlation was observed between the total thickness deterioration rate and dosage of chlorpromazine equivalent.

**TABLE 4:** Total retinal thickness reduction after antipsychotic agent treatment

Variable	Temporal parafoveal region	Foveal region	Nasal parafoveal region
FUSCHSASV	238.7 µm ± 23.7 µm	220.8 µm ± 17.5 µm	264.4 µm ± 10.2 µm
FUSCHMASV	249.5 µm ± 25.1 µm	225.6 µm ± 14.7 µm	274.7 µm ± 10.0 µm
FUSCHSAMV	263.4 µm ± 25.5 µm	221.6 µm ± 21.0 µm	279.1 µm ± 18.9 µm
FUSCHMAMV	242.7 µm ± 18.5 µm	221.9 µm ± 18.0 µm	268.3 µm ± 5.8 µm
Total retinal thickness difference in nasal parafoveal region before and after antipsychotic agents			

	Before treatment	After treatment	*t*	*p*
FUSCHSASV	310.3 µm ± 10.7 µm	264.4 µm ± 10.2 µm	22.50	<.001
FUSCHMASV	312.2 µm ± 14.5 µm	274.7 µm ± 10.0 µm	27.17	<.001
FUSCHSAMV	317.2 µm ± 25.4 µm	279.1 µm ± 18.9 µm	19.65	<.001
FUSCHMAMV	310.5 µm ± 10.7 µm	268.3 µm ± 5.8 µm	18.43	<.001
Total retinal thickness difference in foveal region before and after antipsychotic agents				

	Before treatment	After treatment	*t*	*p*
FUSCHSASV	250.9 µm ± 20.9 µm	220.8 µm ± 17.5 µm	18.54	<.001
FUSCHMASV	253.5 µm ± 15.0 µm	225.6 µm ± 14.7 µm	13.23	<.001
FUSCHSAMV	257.1 µm ± 16.5 µm	221.6 µm ± 21.0 µm	11.20	<.001
FUSCHMAMV	254.5 µm ± 12.0 µm	221.9 µm ± 18.0 µm	11.35	<.001
Total retinal thickness difference in temporal parafoveal region before and after antipsychotic agents				

	Before treatment	After treatment	*t*	*p*
FUSCHSASV	276.0 µm ± 15.0 µm	238.7 µm ± 23.7 µm	15.62	<.001
FUSCHMASV	280.3 µm ± 28.3 µm	249.5 µm ± 25.1 µm	9.85	<.001
FUSCHSAMV	274.0 µm ± 19.5 µm	263.4 µm ± 25.5 µm	8.77	<.001
FUSCHMAMV	275.5 µm ± 10.0 µm	242.7 µm ± 18.5 µm	23.10	<.001

Abbreviations: FUSCHAV, first‐episode untreated schizophrenia with combined AHs and VHs; FUSCHMAMV, middle‐to‐moderate AHs combined with middle‐to‐moderate VHs; FUSCHMASV, middle‐to‐moderate AHs combined with severe VHs; FUSCHSAMV, severe AHs combined with middle‐to‐moderate VHs; FUSCHSASV, severe AHs combined with severe VHs.

## 7 DISCUSSION

The present pilot and secondary follow‐up study investigated the brain and retinal alterations that occurred in FUSCHAV patients. In the pilot study, all four FUSCHAV groups demonstrated GMV reductions, primarily in the temporal, occipital, frontal, and parietal lobes. These findings indicate that the primary auditory and visual cortexes are impaired in FUSCHAV patients (Bernardin et al., 2019; Csaszar, Kapocs, & Bokkon, 2019; Moseley, Mitrenga, Ellison, & Fernyhough, 2018). First‐episode schizophrenia patients with congenital deficiencies or serious brain damage generally have severe AHs and VHs. More notably, the GMV reduction in the frontal and parietal lobes suggests that the cortex involvement affects higher integration. The patients in the current study were experiencing their first episode of schizophrenia and were unmedicated; therefore, our findings suggest that GMV reductions were present prior to the psychotic episode (Bordier, Nicolini, Forcellini, & Bifone, 2018; Zmigrod et al., 2016) and that there may be a brain‐related pathology associated with the onset of AHs and VHs. Moreover, the FUSCHAV patients demonstrated increased gFCD in the same brain regions showing GMV reductions, suggesting that functional hyperactivity may compensate for structural impairment (Cao et al., 2018; Collier et al., 2014; Csaszar et al., 2019; Kremlacek et al., 2016; Xu, Qin, Liu, Jiang, & Yu, 2016). While some brain regions demonstrated an increase in the GMV and gFCD between the different FUSCHAV groups, these alterations may be distinct to each patient group. In addition to alterations in the brain gray matter and connectivity, the total retinal thickness was decreased in the FUSCHAV patients compared to that in the healthy controls, suggesting the presence of brain and eye co‐impairment.

The pilot study yielded three major findings. First, the GMV reduction and gFCD increase in the frontal‐parietal lobe of the FUSCHAV patients demonstrated an inverted U‐shaped pattern according to the AH and VH severity combination. This inverted U‐shaped pattern suggests that visual disturbances are accompanied by relatively severe brain structural impairments and relatively strong functional compensation. However, the GMV alterations in the FUSCHSASV group were more severe than those in the FUSCHSAMV group, indicating that the severity of VHs may enhance brain structural impairment and functional compensation in the FUSCHSASV group. The second major finding was that auditory and visual disturbances may be a result of reciprocal deterioration in such patients, as reflected in the inverted U‐shaped pattern of GMV and gFCD. These findings provide new clues for the further exploration of the mechanisms of the interaction between AHs and VHs in schizophrenia. Third, although the brain alterations in the FUSCHAV patients demonstrated an inverted U‐shaped pattern, according to the severity of the AHs and VHs, the degree of retinal impairment in these patients did not differ. This finding suggests that, while the different severity combinations of AHs and VHs are related to brain alterations, retinal thickness may be more of a trait marker and not related to symptom severity. This idea is consistent with that observed in previous studies conducted in first‐episode unmedicated patients (Bernardin et al., 2019; Moseley et al., 2018; Thomas, 1990).

The most important finding of the secondary follow‐up study was that the effect of antipsychotic agent treatment for 6 months in all the four patient groups was very unsatisfactory. The PANSS scores reduced rate in any patient group can not be reach 50% or above. These findings demonstrate that regular treatment yields only weak effects, indicating that such patients are at a high risk of developing treatment‐resistant schizophrenia (Gillespie, Samanaite, Mill, Egerton, & MacCabe, 2017; Kane et al., 2019).

Second, the GMV deteriorated in all the patients. The extent of this deterioration did not demonstrate an inverted U‐shaped pattern, and the compensatory increase in the gFCD of all the patients was reversed to a gFCD decrease with antipsychotic agent use. The brain regions with GMV deterioration and gFCD decreases did not cope with those with GMV impairment and compensatory gFCD increases at the baseline. Both the GMV deteriorations and gFCD decreases were located in the occipital, parietal, temporal, and frontal lobes. These findings indicate that antipsychotic agent treatment induces brain structural and functional alterations at almost all the pivotal regions required for cognitive function, executive function, and mood regulation networks/circuits (Cao et al., 2018; Collier et al., 2014; Csaszar et al., 2019; Kremlacek et al., 2016; Xu et al., 2016), consistent with the findings of previous studies, which reported that retinal thickness and brain GMV reductions are affected by treatment with antipsychotics (Bordier et al., 2018; Cao et al., 2018; Collier et al., 2014; Csaszar et al., 2019; Kremlacek et al., 2016; Xu et al., 2016; Zmigrod et al., 2016). Fortunately, we did not observe a GMV reduction rate greater than 2% in all the patients. Some previous studies have reported that GMV reductions predominantly occur in the 2–3 years after treatment and did not demonstrate a continued deterioration tendency (Hulshoff Pol & Kahn, 2008; Kuo & Pogue‐Geile, 2019; Palaniyappan et al., 2015; Smieskova et al., 2009; Vita, De Peri, Deste, & Sacchetti, 2012); the results of our secondary follow‐up study support those of previous studies.

Third, we observed total retinal thickness deteriorations as a consequence of antipsychotic agent treatment. The deterioration extent was complex and did not cope with the values at the baseline. The maximum deterioration rates were 6.7% on self‐comparison at the baseline and 21.8% (compared to that in the healthy controls at the baseline). These findings indicate that antipsychotic agent treatment leads to retinal thickness deterioration. However, the retinal thickness deterioration tendency has not been reported on in previous studies; hence, further long‐term studies with a duration longer than 2 years are needed to clarify the trajectory of retinal thickness in patients with FUSCHAV.

### 7.1 Limitations

There are several limitations to the current pilot and secondary follow‐up study that must be considered in result interpretation. First, the secondary follow‐up study was conducted based on the findings of the pilot study; the validity of this method has not been confirmed. Further studies must be performed to test the validity of this method. Second, in the secondary follow‐up study, we observed brain and retinal deteriorations after 6 months; it is of concern that 6 months may not be sufficient in confirming a patient's brain and retinal deterioration tendency; therefore, long‐term (2–3 years) studies are needed to further clarify this point. Third, in this pilot and secondary study, we only selected patients with FUSCHAV, due to which sample bias cannot be avoided; studies including large numbers of schizophrenia patients without AH and VH symptoms are required to negate the effects of sample bias. Fourth, in the secondary follow‐up study, the antipsychotic agent treatment strategy and dosage were changed in some patients; this may have led to confounding, although we regressed out the dosage effect. Fifth, the absence of a group of schizophrenia patients without AHs and VHs weakened the validity of our findings and conclusions, although schizophrenia patients without AHs and VHs are very difficult to recruit; future studies should include this group to enhance the strength of the conclusions. Sixth, the small sample size of each group limits the significance of the findings. Seventh, the patients were artificially divided into four categories, and this method requires further study for validity testing. Eighth, total retinal thickness was used to assess eye impairment which was limited by the use of OCT‐4000. The retina was not divided into ten layers for the precise clarification of retinal impairment, and, as such, may not provide the full picture of retinal impairment in FUSCHAV. Ninth, the correlations between the GMV, we observed the gFCD, retinal thickness, antipsychotic agent dosage, and clinical symptoms were not significantly in our pilot and secondary follow‐up study. These findings weaken our conclusions, which state that antipsychotic agent treatment is accompanied by deteriorated GMV and retinal thickness. Future large‐sample, long‐term cohort studies should be conducted to enhance the strength of our conclusions.

## 8 CONCLUSION

Despite the above‐described limitations, our findings indicate the need for heightened alertness on brain and retinal impairments in FUSCHAV, and monitoring for the further deterioration induced by antipsychotic agent treatment in clinical practice.

## CONFLICT OF INTEREST

None declared.

## AUTHORS CONTRIBUTIONS

CZhuo, XB, CC and XL conceived and designed research; CC, XB, DJ, and GL collected data and conducted research; XM, RL, and LW analyzed and interpreted data; YX and CZhou wrote the initial paper; CZhuo and CZhou revised the paper; XL had primary responsibility for final content. All authors read and approved the final manuscript.

## Data Availability

The datasets generated and analyzed during the present study are available from the corresponding author on reasonable request.
